# Long non-coding RNA MEG3 functions as a competing endogenous RNA to regulate ischemic neuronal death by targeting miR-21/PDCD4 signaling pathway

**DOI:** 10.1038/s41419-017-0047-y

**Published:** 2017-12-13

**Authors:** Honglin Yan, Jie Rao, Jingping Yuan, Likun Gao, Wenxian Huang, Lina Zhao, Jiacai Ren

**Affiliations:** Department of Pathology, Renmin Hospital of Wuhan University, Wuhan, 430060 China

## Abstract

Long non-coding RNA (lncRNA) maternally expressed gene 3 (MEG3) has been demonstrated as an important regulator in diverse human cancers. However, its function and regulatory mechanism in ischemic stroke remains largely unknown. Here, we report that MEG3 is physically associated with microRNA-21 (miR-21), while miR-21 is downregulated following ischemia in the ischemic core *in vitro* and* in vivo*, which is opposite to MEG3. Besides, overexpression of miR-21 protects oxygen–glucose deprivation and reoxygenation (OGD/R)-induced apoptotic cell death. Furthermore, MEG3 functions as a competing endogenous RNAs (ceRNAs) and competes with programmed cell death 4 (PDCD4) mRNA for directly binding to miR-21, which mediates ischemic neuronal death. Knockdown of MEG3 protects against ischemic damage and improves overall neurological functions* in vivo*. Thus, our data uncovers a novel mechanism of lncRNA MEG3 as a ceRNA by targeting miR-21/PDCD4 signaling pathway in regulating ischemic neuronal death, which may help develop new strategies for the therapeutic interventions in cerebral ischemic stroke.

## Introduction

Stroke is an acute cerebrovascular disease caused by poor blood flow to the brain. Approximately 85% of all reported strokes are due to cerebral ischemia which occurs when an embolus or thrombus blocks the major cerebral artery resulting in cell death^1^. At present, the only effective treatment of ischemic stroke is the application of tissue plasminogen activator (tPA)^2^. However, due to the narrow therapeutic window (less than 4.5 h), strict indications for treatment and the risk of secondary hemorrhage, only a few stroke sufferers benefit from it. Thus, failures in recovery of blood flow highlights the need for an alternative method for the treatment of cerebral ischemia^3^. During the first 3 days following cerebral ischemia, the secondary brain injury progresses rapidly and then at a slower pace up to 2 weeks^4^. Studies have documented that the secondary neuronal death is the major cause of cerebral infarction, leading to a long-term neurological deficits after stroke^3,5^. As a series of complex biochemical incidents including excitotoxicity, oxidative stress, ionic imbalance, endoplasmic reticulum stress and inflammation following cerebral ischemia lead to cell death and produce profound neuronal damage, more evidence emphasizes the importance of exploring the nature of ischemic neuronal death^3,6–8^.

Over the last decade, advances in genome-wide sequencing technologies have revealed that non-coding transcripts dominate genomes transcriptional output^9,10^. In the brain’s responses to ischemia, dysregulation of non-coding RNAs (ncRNAs) has been closely related to neurologic functional disorders and the expression of various key elements in cell death^11^. Accumulative evidence has demonstrated that cerebral ischemia significantly changes the expression profiles of multiple ncRNA species, including long non-coding RNAs (lncRNAs, >200 nt), microRNAs (miRNAs, 20–25 nt), and piwi-interacting RNAs (piRNAs)^5,12–15^. Among these RNAs, miRNAs are the most studied of all ncRNAs as critical mediator of ischemia^4,5^. Unlike the miRNAs, the function of lncRNAs in the pathologies of stroke is poorly understood^5^. Importantly, increasing experimental evidence has confirmed that lncRNAs act as competing endogenous RNAs (ceRNAs) by competing for binding to miRNAs, and this crosstalk have been implicated in various biological processes in human development and disease^16–18^. Additionally, miRNAs are involved in a variety of biological processes by modulating their target mRNAs. Hence, it is possible that the interaction between lncRNA–miRNA–mRNA plays an critical role in the development of ischemia.

Maternally expressed gene 3 (MEG3), also known as gene trap locus 2 (Gtl2), is an imprinted gene located on chromosome 14q32.3 in humans and functions as a lncRNA^19^. Since the loss of MEG3 has been reported in various types of human cancers, including gastric, prostate, hepatocellular, breast, and liver cancers, more previously research focused on MEG3 as a tumor suppressor in cell proliferation^20–23^. However, less is known regarding the biology and function of MEG3 in ischemia. Our previous study observed that ischemia altered cerebral MEG3 profiles *in vitro* and *in vivo* ^24^. Furthermore, MEG3 interacted with p53 and activated p53-mediated transactivation to mediate ischemic neuronal death in stroke^24^. Thus, the increased expression of MEG3 in ischemia highlights its role as an emerging target in the treatment of ischemia. Nevertheless, it is not clear whether MEG3 functions as a ceRNA competing for binding to miRNAs in the development of ischemia.

In the current study, we first determined the functional interaction among MEG3, microRNA-21 (miR-21), and programmed cell death 4 (PDCD4) in ischemia. We found MEG3 was physically associated with miR-21, while miR-21 was downregulated following ischemia in the ischemic core *in vitro* and *in vivo*. We further confirmed that the overexpression of miR-21 *in vitro* protected oxygen–glucose deprivation/reoxygenation (OGD/R)-induced neuronal death and this effect could be abolished through the use of miR-21 inhibitor. Moreover, MEG3 competed with PDCD4 mRNA for binding to miR-21 and contradicted the inhibitory effects of miR-21 on PDCD4 to regulate ischemic neuronal death. Knockdown of MEG3 protected against I/R-induced ischemic brain damage and improved overall neurological functions. These findings indicate that lncRNA MEG3 functions as a ceRNA for miR-21 to regulate PDCD4 expression in ischemic neuronal death followed by reperfusion, which may inform new therapeutic strategies for ischemic insults.

## Results

### MEG3 is physically associated with miR-21

**Fig. 1:**
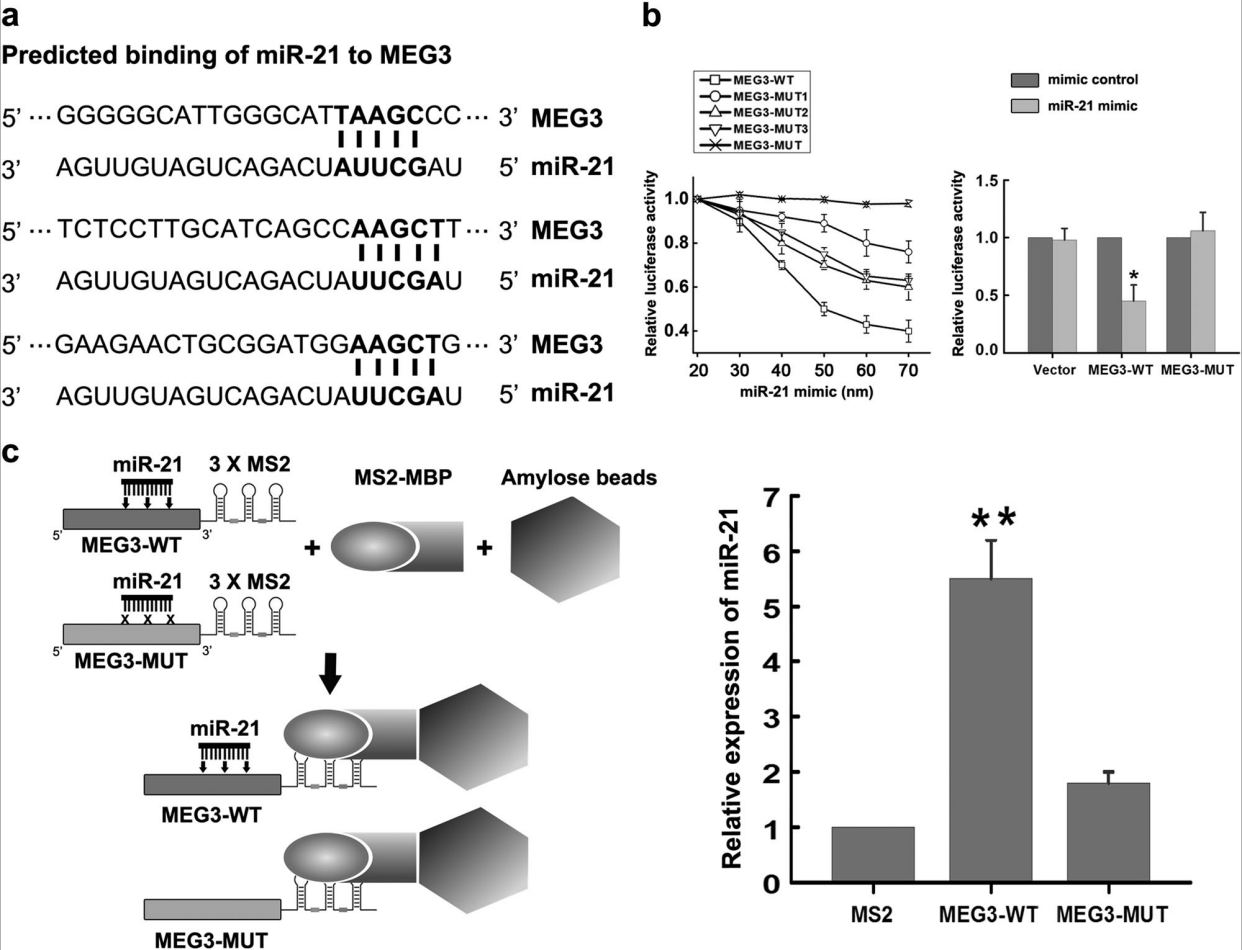
The interaction of MEG3 with miR-21 **a** The prediction for MEG3 transcript binding sites on miR-21 by bioinformatics analysis. The nucleotides in bold are the complementary sequences to miR-21 seed sequences. **b** MEG3 is physically associated with miR-21. Curve lines (left) summarize the relative luciferase reporter activities with the increase of miR-21 concentration. Bar graph (right) shows relative luciferase activities in N2a cells cotransfected with miR-21 mimic (50 nM) + Vector, or miR-21 mimic (50 nM) + MEG3-WT, or miR-21 mimic (50 nM) + MEG3-MUT, or mimic control (50 nM) + Vector, or mimic control (50 nM) + MEG3-WT, or mimic control (50 nM) + MEG3-MUT. Data are means ± S.E.M. for 3 independent experiments. ^*^ *P* < 0.05 by Student’s *t*-test. Vector, luciferase reporters containing nothing; MEG3-WT, luciferase reporters containing the wild type MEG3; MEG3-MUT, luciferase reporters containing the mutant MEG3 with mutations of all three predicted miR-21-binding sites. **c** RNA pulldown assay based on MS2-MBP followed by miRNA qRT-PCR to detect miR-21 endogenously associated with MEG3. The schematic diagram on the left shows the strategies of the experiment. Bar graph (right) summarizes the relative expression of miR-21 in cells transfected with MEG3-WT-MS2 (MEG3-WT), MEG3-MUT-MS2 (MEG3-MUT) or the empty vector (MS2). Data are means ± S.E.M. for 3 independent experiments. ^**^ *P* < 0.01 by Student’s *t*-test

According to the recent study, ceRNAs, such as lncRNAs, could compete for binding to miRNAs through miRNA response elements (MREs), thus preventing the binding of miRNAs to target mRNAs^25^. Our previous study has revealed that lncRNA MEG3 functioned as a cell death promoter in ischemia^24^. However, the mechanisms underlying the effects of MEG3 in ischemia are not well understood. So we proposed that MEG3 may function as a ceRNA to regulate miRNAs. Sequence analysis and an open online database lnCeDB provided 3 putative-binding sites between MEG3 and miR-21 (Fig. 1a), suggesting its ceRNA potential for miR-21. Subsequently, we constructed luciferase reporters containing the wild type MEG3 (pmirGLO-MEG3-WT), or mutant MEG3 with mutations of single (pmirGLO-MEG3-MUT1, 2, 3). We also overexpressed miR-21 via various concentrations of miR-21 mimic. We found the affinity of diverse mutant MEG3 (pmirGLO-MEG3-MUT1, 2, 3) to miR-21 was different. With the increase of miR-21 concentration, it showed the least decrease in luciferase reporter activities of the pmirGLO-MEG3-MUT1 (Fig. 1b, left), which suggested that the first putative-binding sites between MEG3 and miR-21 in Fig. 1a were more important in determining the affinity of MEG3 to miR-21. To maximize the mutation efficiency, mutant MEG3 with mutations of all three predicted miR-21-binding sites (pmirGLO-MEG3-MUT) was also constructed. However, the luciferase reporter activities of the pmirGLO-MEG3-MUT reporter did not change with the increase of miR-21 (Fig. 1b, left). Moreover, compared to mimic control, miR-21 mimic remarkably reduced the luciferase reporter activities of the pmirGLO-MEG3-WT reporter, but not that of the pmirGLO-MEG3-MUT reporter with mutations of all three predicted miR-21-binding sites (Fig. 1b, right), suggesting that MEG3 was physically associated with miR-21 via these sites. To further validate the direct interaction between miR-21 and MEG3, we transfected N2a cells with MS2-containing MEG3 (MEG3-WT-MS2) constructs and performed an RNA pulldown assay based on MS2-maltose-binding protein (MS2-MBP) to identify endogenous miRNAs that associated with MEG3. Quantitative real-time PCR (qRT-PCR) analysis of the precipitated miRNAs showed that MS2-containing MEG3 with mutations in all three miR-21-targeting sites (MEG3-MUT-MS2) was significantly enriched for miR-21 compared to the empty vector (MS2) (Fig. 1c). It supported that miR-21 was bona fide MEG3-targeting miRNA.

### miR-21 is downregulated following ischemia *in vitro* and *in vivo*

**Fig. 2:**
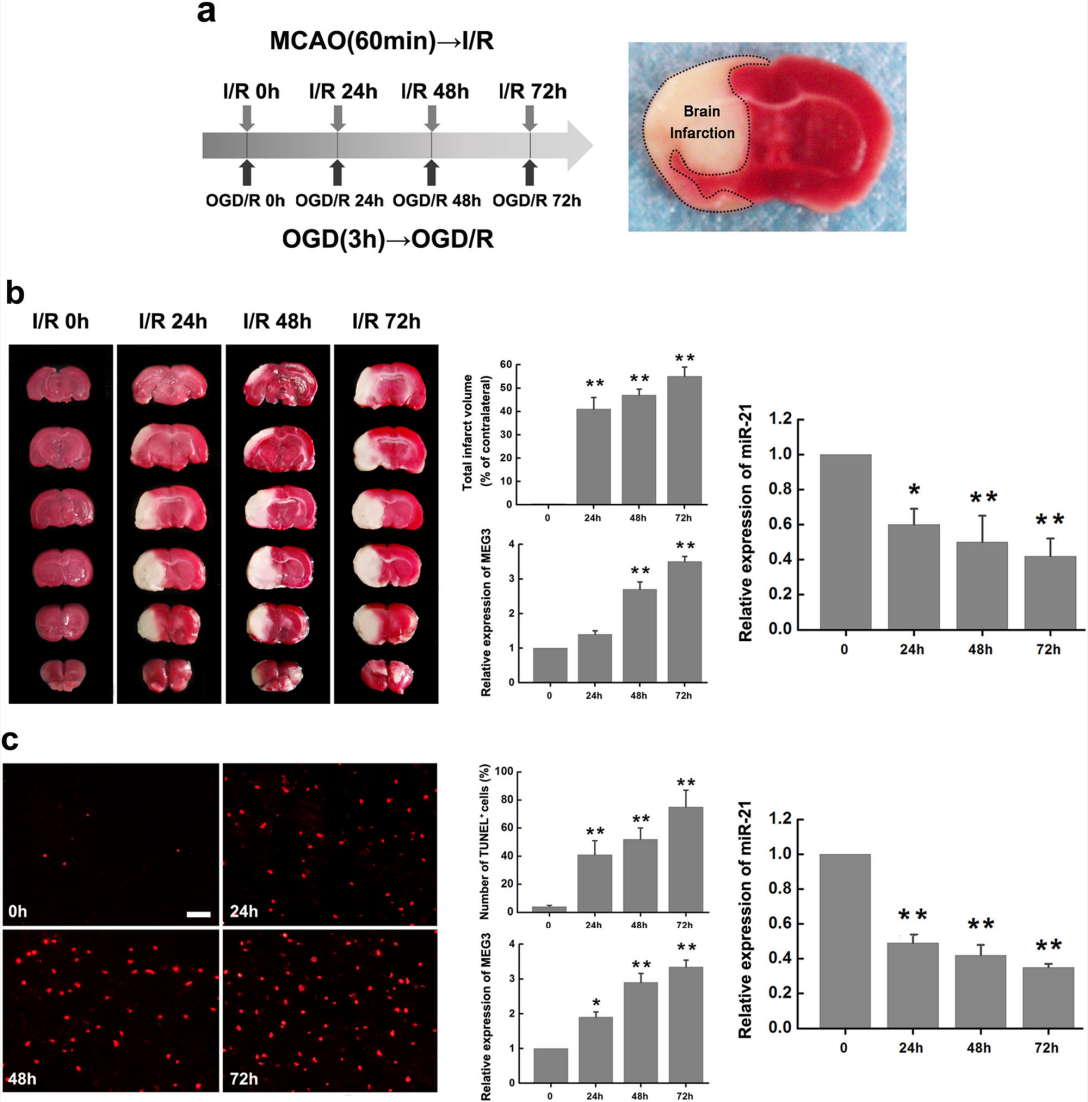
miR-21 is downregulated following ischemia *in vitro* and *in vivo* **a** Illustration shows the experimental procedure of ischemic model* in vitro* (OGD/R) and *in vivo* (I/R). Focal ischemia induced by MCAO operation resulted in brain damage that was visible in TTC-stained brain section (right). The brain infarction area is marked. **b** Focal ischemia induced by MCAO resulted in increased brain damage with reperfusion time following I/R (left). Bar graph (middle and upper part) shows the volumes of total cerebral infract in ipsilateral hemisphere normalized to the total volumes of the contralateral hemisphere. Bar graph (middle and lower part) and bar graph (right) show relative expression of MEG3 and miR-21 in the ischemic core at different time points following I/R. Data are means ± S.E.M. for 6 mice per group. ^*^ *P* < 0.05, ^**^ *P* < 0.01, by Student’s *t*-test. **c** Apoptotic cell death of N2a cells challenged with OGD was measured by TUNEL staining at different time points of reoxygenation. Bar graph (middle and upper part) shows the number of TUNEL^+^ cells in different groups. Bar graph (middle and lower part) and bar graph (right) show relative expression of MEG3 and miR-21 at different time points following OGD/R. Scale bar, 25μm. Data are means ± S.E.M. for 5 cultures per group. *P < 0.05, ^**^ *P* < 0.01, by Student’s *t*-test

Based on above results, miR-21 may also play a vital role in ischemic stroke. To evaluate the role of miR-21 in neuronal death following ischemia and reperfusion (I/R) *in vivo*, focal cerebral ischemia was induced in adult male mice by middle cerebral artery occlusion (MCAO) for 60 min, followed by a reperfusion for 24, 48, or 72 h, as outlined in Fig. 2a. Ischemic lesions were detected by 2,3,5-triphenyltetrazolium chloride (TTC) staining, while focal ischemia caused cell death in the brain infarction was unstained (Fig. 2a, right). Focal ischemia induced by MCAO operation resulted in increased brain damage with reperfusion time following I/R (Fig. 2b, left). Infarction tissues in the ischemic core were isolated post model establishment for qRT-PCR assay. In contrast to the consistently upregulation of MEG3 following ischemia (Fig. 2b, middle), miR-21 was abruptly decreased at 24 h of reperfusion, and then downregulated at a much slower pace at 48 h and 72 h in the ischemic core, indicating its potential role in ischemia (Fig. 2b, right). Then, N2a cell oxygen–glucose deprivation and reoxygenation (OGD/R) model was established to further determine the expression of miR-21 in response to ischemia *in vitro* (Fig. 2a). As reported^1^, TUNEL assay indicated that OGD/R-induced apoptotic cell death gradually increased with reoxygenation time (Fig. 2c, left). Besides, OGD/R treatment caused the same expression trend of MEG3 and miR-21 in N2a cell as MCAO mice at different time points of reoxygenation, in which showed time-dependent upregulation of MEG3 (Fig. 2c, middle) and downregulation of miR-21 (Fig. 2c, right). Therefore, miR-21 is downregulated following ischemia *in vitro* and *in vivo*.

### miR-21 protects OGD/R-induced neuronal death

**Fig. 3:**
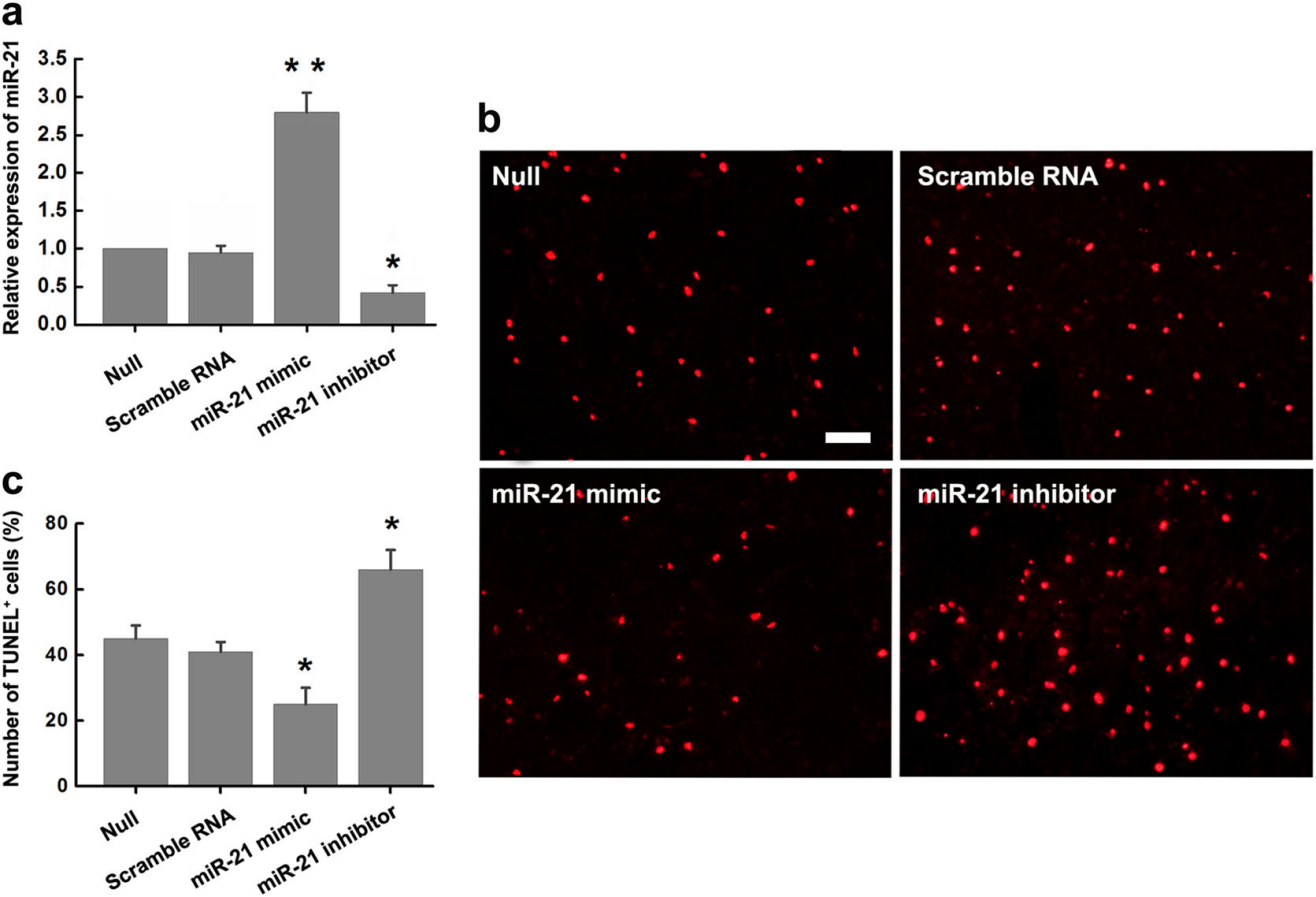
miR-21 protects OGD/R-induced neuronal death **a** The modulation of miR-21 levels by scramble RNA, miR-21 mimic or miR-21 inhibitor in N2a cells were confirmed by qRT-PCR. Data are means ± S.E.M. for 3 independent experiments. ^*^ *P* < 0.05, ^**^ *P* < 0.01, by Student’s *t*-test. **b** Apoptotic cell death of N2a cells transfected with scramble RNA, miR-21 mimic or miR-21 inhibitor was measured by TUNEL staining at 24 h after OGD treatment. Scale bar, 25μm. **c** Bar graph summarizes the number of TUNEL^+^ cells in different groups. Data are means ± S.E.M. for 5 cultures per group. ^*^ *P* < 0.05 by Student’s *t*-test

Considering the consistently downregulation of miR-21 in response to ischemia *in vitro* and *in vivo*, we next examined the involvement of miR-21 in OGD/R-induced neuronal cell death by artificially overexpressed miR-21 via miR-21 mimic or knocked it down by miR-21 inhibitor. Thus, N2a cells were transfected with miR-21 mimic, miR-21 inhibitor, or a negative control of scramble RNA. qRT-PCR was used to measure the expression of miR-21 after transfection. It showed that miR-21 expression was increased ~3-fold in miR-21 mimic transfected cells and reduced to ~40% in miR-21 inhibitor-transfected cells (Fig. 3a). Three days after transfection, we challenged N2a cells with OGD for 3 h, followed by a reoxygenation for 24 h. Accordingly, TUNEL staining showed that overexpression of miR-21 significantly abolished OGD/R-induced apoptotic cell death as compared with scrambled control treatment (Fig. 3b, c). Whereas, more TUNEL-positive cells were observed when inhibition of endogenous miR-21 by miR-21 inhibitor (Fig. 3b, c). Taken together, miR-21 protects OGD/R-induced neuronal death *in vitro*, which is opposite to MEG3.

### MEG3 competes with PDCD4 mRNA for binding to miR-21

**Fig. 4:**
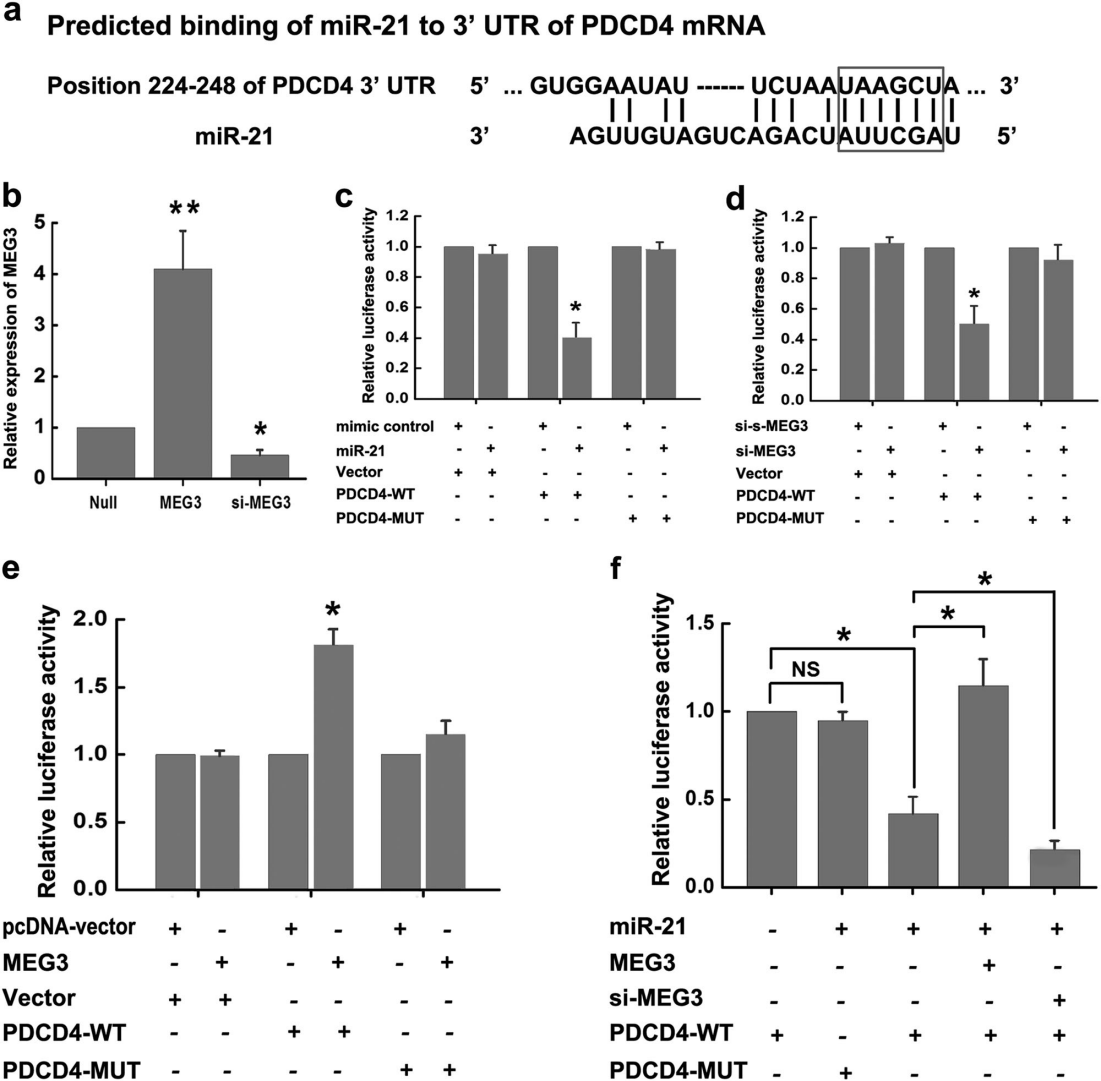
MEG3 competes with PDCD4 mRNA for binding to miR-21 **a** miR-21 bounds to 3’-UTR of PDCD4 mRNA by bioinformatics analysis. Overlapping sequences of the binding sites of MEG3–miR-21 and PDCD4–miR-21 are marked with a rectangular box. **b** The modulation of MEG3 levels by MEG3-expressing plasmids or si-MEG3 in N2a cells were confirmed by qRT-PCR. Data are means ± S.E.M. for 3 independent experiments. ^*^ *P* < 0.05, **P < 0.01, by Student’s *t*-test. **c** N2a cells were cotransfected with mimic control + Vector, or miR-21 + Vector, or mimic control + PDCD4-WT, or miR-21 + PDCD4-WT, or mimic control + PDCD4-MUT, or miR-21 + PDCD4-MUT. Relative luciferase activity was measured by luciferase reporter assays. Data are means ± S.E.M. for 3 independent experiments. ^*^ *P* < 0.05 by Student’s *t*-test. miR-21, miR-21 mimic; Vector, luciferase reporters containing nothing; PDCD4-WT, luciferase reporters containing wild type PDCD4; PDCD4-MUT, luciferase reporters containing mutant PDCD4. **d** N2a cells were cotransfected with si-s-MEG3 + Vector, or si-MEG3 + Vector, or si-s-MEG3 + PDCD4-WT, or si-MEG3 + PDCD4-WT, or si-s-MEG3 + PDCD4-MUT, or si-MEG3 + PDCD4-MUT. Relative luciferase activity was measured by luciferase reporter assays. Data are means ± S.E.M. for 3 independent experiments. ^*^ *P* < 0.05 by Student’s *t*-test. si-s-MEG3, negative control of scramble RNA for si-MEG3; si-MEG3, MEG3-targeting small interference RNAs. **e** N2a cells were cotransfected with pcDNA-vector + Vector, or MEG3 + Vector, or pcDNA-vector + PDCD4-WT, or MEG3 + PDCD4-WT, or pcDNA-vector + PDCD4-MUT, or MEG3 + PDCD4-MUT. Relative luciferase activity was measured by luciferase reporter assays. Data are means ± S.E.M. for 3 independent experiments. ^*^ *P* < 0.05 by Student’s *t*-test. pcDNA-vector, pcDNA3.0 empty vector which was used to MEG3-expressing plasmids construction; MEG3, MEG3-expressing plasmids; **f** N2a cells were cotransfected with PDCD4-WT, or miR-21 + PDCD4-MUT, or miR-21 + PDCD4-WT, or miR-21 + MEG3 + PDCD4-WT, or miR-21 + si-MEG3 + PDCD4-WT. Relative luciferase activity was measured by luciferase reporter assays. Data are means ± S.E.M. for 3 independent experiments. ^*^ *P* < 0.05 by Student’s *t*-test. NS, not significant

The protection role of miR-21 in ischemic neuronal death promoted us to explore its mechanism in ischemia. Bioinformatics analysis using TargetScan indicated that miR-21 bounds to 3′-UTR of PDCD4 mRNA (Fig. 4a). By comparing the binding sites of miR-21–PDCD4 and MEG3–miR-21, we found that there were some overlapping sequences (marked with a rectangular box in Fig. 4a), suggesting that MEG3 may affect the binding between miR-21 and 3′-UTR of PDCD4 mRNA. Next, we determined the interaction among MEG3, miR-21, and PDCD4. We first prepared miR-21 mimic, MEG3-targeting small interference RNAs (si-MEG3), negative control of scramble RNA for si-MEG3 (si-s-MEG3), and constructed a series of plasmids, such as MEG3-expressing plasmids, luciferase reporters containing the wild type PDCD4 with 3′-UTR (pmirGLO-PDCD4-WT), or a mutant PDCD4 with mutations at the binding sites between 3′-UTR of PDCD4 mRNA and miR-21 (pmirGLO-PDCD4-MUT). Then, the modulation of MEG3 levels by MEG3-expressing plasmids or si-MEG3 in N2a cells were confirmed by qRT-PCR (Fig. 4b). After that, luciferase reporter assays were performed. We found that the miR-21 mimic significantly decreased the luciferase activities of pmirGLO-PDCD4-WT reporter, but not of pmirGLO-PDCD4-MUT reporter (Fig. 4c). Similar results were also obtained in cells cotransfected si-MEG3 with pmirGLO-PDCD4-WT (Fig. 4d). In contrast, overexpression of MEG3 increased the luciferase activities of pmirGLO-PDCD4-WT, but not of pmirGLO-PDCD4-MUT (Fig. 4e). On the other hand, the suppressive effects of miR-21 on the luciferase activity of pmirGLO-PDCD4-WT could be abolished by overexpression of MEG3, while augmented by si-MEG3 (Fig. 4f). Thus, all these data indicated that MEG3 competes with PDCD4 mRNA for binding to miR-21.

### MEG3 contradicts the inhibitory effects of miR-21 on PDCD4 to regulate ischemic neuronal death

**Fig. 5:**
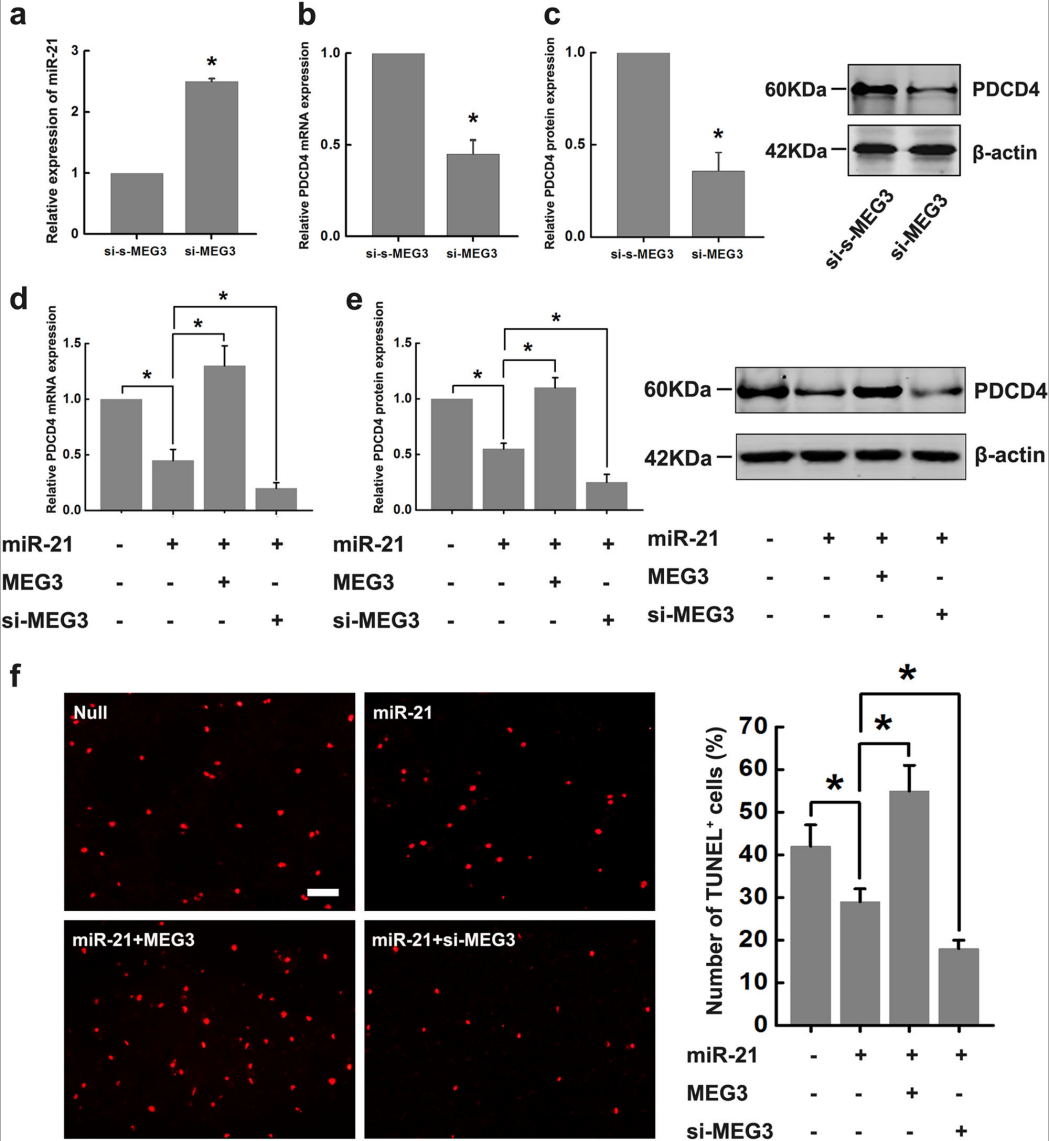
MEG3 contradicts the inhibitory effects of miR-21 on PDCD4 to regulate ischemic neuronal death **a**–**c** The modulation of miR-21 (**a)**, PDCD4 mRNA (**b)** and protein (**c)** levels by transfection of si-MEG3 in N2a cells were confirmed by qRT-PCR and western blot. Data are means ± S.E.M. for 3 independent experiments for all 3 parameters. ^*^ *P* < 0.05 by Student’s *t*-test. (**d** and **e**) Relative expression of PDCD4 mRNA (**d)** and protein (**e)** in N2a cells transfected with miR-21, or miR-21 + MEG3, or miR-21 + si-MEG3. Data are means ± S.E.M. for 3 independent experiments for the 2 parameters. ^*^ *P* < 0.05 by Student’s *t*-test. **f** Apoptotic cell death of N2a cells transfected with miR-21, or miR-21 + MEG3, or miR-21 + si-MEG3 was measured by TUNEL staining at 24 h after OGD treatment. Bar graph (right) shows the number of TUNEL^+^ cells in different groups. Scale bar, 25μm. Data are means ± S.E.M. for 5 cultures per group. ^*^ *P* < 0.05 by Student’s *t*-test

Based on the above results, we hypothesized that MEG3 affects PDCD4 expression through downregulation of the miR-21 level. As we expect, the upregulation of miR-21 (Fig. 5a), downregulation of PDCD4 mRNA (Fig. 5b) and protein (Fig. 5c) were observed in the si-MEG3 transfected cells. So we next examined the N2a cells transfected with miR-21 mimic, or miR-21 mimic + MEG3-expressing plasmids, or miR-21 mimic + si-MEG3 for PDCD4. It showed that miR-21 significantly decreased the expression of PDCD4 mRNA and protein (Fig. 5d, e). However, the effects of miR-21 on PDCD4 were abolished by MEG3 overexpression, and augmented by si-MEG3, further supporting the luciferase reporter assays (Fig. 5d, e). Moreover, compared to the cells transfected with miR-21 mimic and challenged with OGD/R, it showed more TUNEL-positive cells when cotransfection of miR-21 mimic + MEG3-expressing plasmid, while less TUNEL-positive cells when cotransfection of miR-21 mimic + si-MEG3, indicating the protection effect of miR-21 on OGD/R-induced neuronal death were abolished by MEG3 overexpression, and augmented by si-MEG3 (Fig. 5f). These series of results indicated that MEG3 regulates ischemic neuronal death by contradicting the inhibitory effects of miR-21 on PDCD4.

### Knockdown of MEG3 protects against ischemic damage and improves neurological deficits *in vivo*

**Fig. 6:**
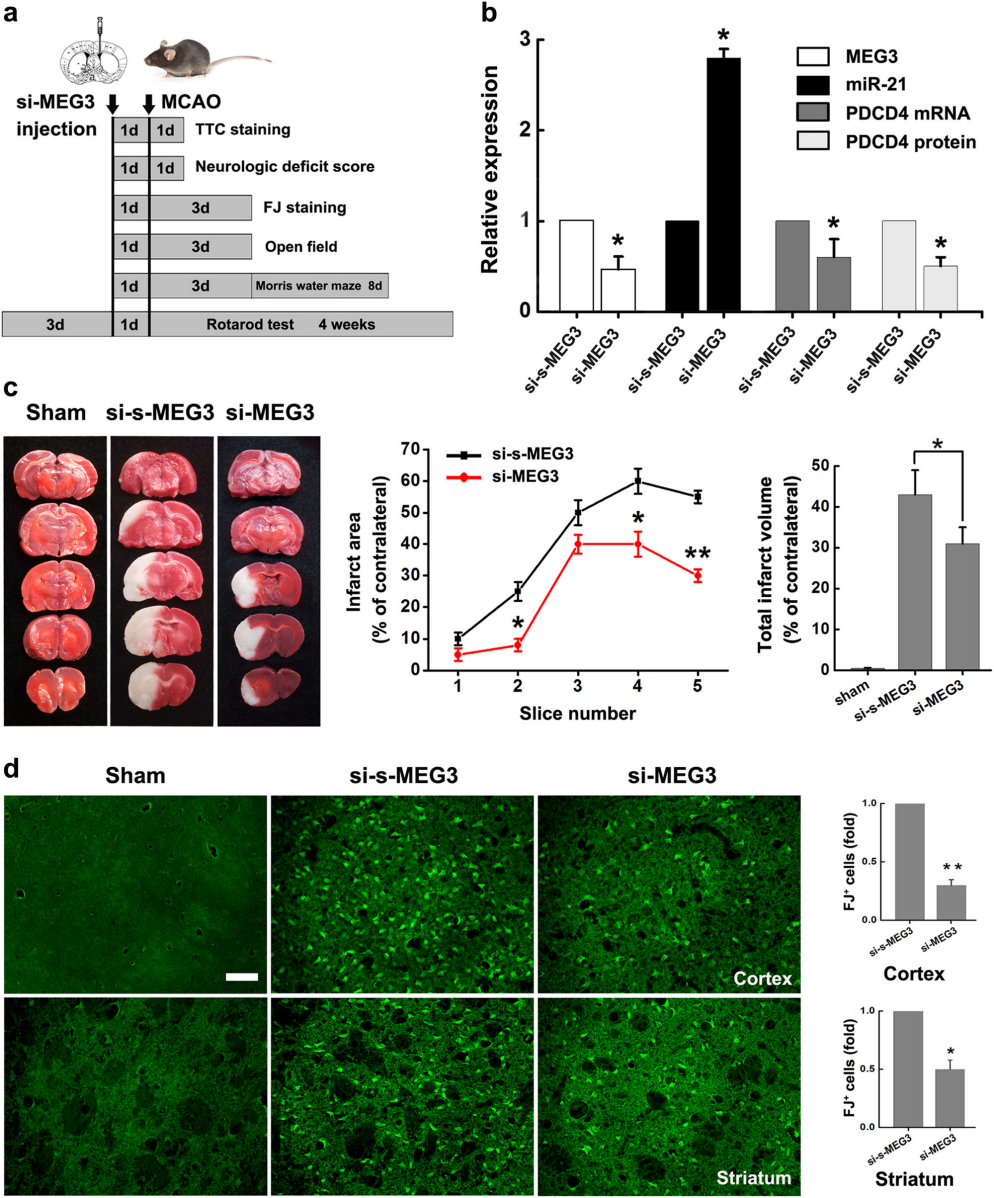
Knockdown of MEG3 protects against I/R-induced ischemic brain damage *in vivo* **a** Experimental schedule to explore the effect of si-MEG3 on ischemic brain damage and overall neurological functions *in vivo*. **b** The modulation of MEG3, miR-21, PDCD4 mRNA and protein levels by injection of si-MEG3 or si-s-MEG3 into the lateral ventricle of mice were confirmed by qRT-PCR and western blot 24 h after the injection. Data are means ± S.E.M. for 3 independent experiments for all 4 parameters. ^*^ *P* < 0.05 by Student’s *t*-test. **c** Brain infarction of three groups (sham-treated mice, si-s-MEG3 and si-MEG3 injection mice subjected to MCAO operation) was visualized in TTC-stained brain sections at 24 h after operation. Curve lines (middle) summarize infarct areas in ipsilateral hemisphere normalized to the total areas of the contralateral hemisphere in sequential coronal brain slices. Bar graph (right) shows the volumes of total cerebral infract in ipsilateral hemisphere normalized to the total volumes of the contralateral hemisphere. Data are means ± S.E.M. for 6 mice per group. ^*^ *P* < 0.05, ^**^ *P* < 0.01, by Student’s *t*-test. **d** Degenerated cells in the cortex and striatum of si-s-MEG3 and si-MEG3 injection mice subjected to I/R 72 h were assessed by FJ staining. Bar graph (right) shows the FJ-labeling cells in the cortex and striatum in si-MEG3 treated mice normalized to si-s-MEG3 treated mice. Scale bar, 50*μ*m. Data are means ± S.E.M. for 6 mice per group. ^*^ *P* < 0.05, ^**^ *P* < 0.01, by Student’s *t*-test

Given that MEG3 functions as a ceRNA to regulate neuronal death by competing with PDCD4 mRNA for binding to miR-21, we hope to further explore the significance of the association. Towards this end, we injected si-MEG3 or si-s-MEG3 with *in vivo* transfection reagent into the lateral ventricle of mice before MCAO operation (Fig. 6a). To validate the expression of MEG3, miR-21, PDCD4 was modulated by injection of si-MEG3 or si-s-MEG3, qRT-PCR and western blot was performed 24 h after si-s-MEG3/si-MEG3 injection. As expected, we observed the upregulation of miR-21, downregulation of MEG3 and PDCD4 in mice injected with si-MEG3 compared with si-s-MEG3 (Fig. 6b). Next, we examined whether knockdown of MEG3 in brains protected ischemic cerebral infarction in mice responding to I/R (Fig. 6a). One day after injection, MCAO operation was established (Fig. 6a). 24 h after reperfusion, the brain infarction was detected by TTC staining to visualize the damaged regions for determination of the infarct volume of I/R injury. It showed that si-MEG3 treatment significantly improved I/R-induced brain infract volume compared with si-s-MEG3 (Fig. 6c). 3 days after reperfusion, neuronal death was detected by Fluoro-Jade C (FJ) staining in frozen brain sections. Much less neuronal death was detected in the vulnerable brain regions including the cortex and the striatum in mice injected with si-MEG3 compared with si-s-MEG3 (Fig. 6d). These results indicated that si-MEG3 were protective against I/R-induced ischemic damage *in vivo*.

**Fig. 7:**
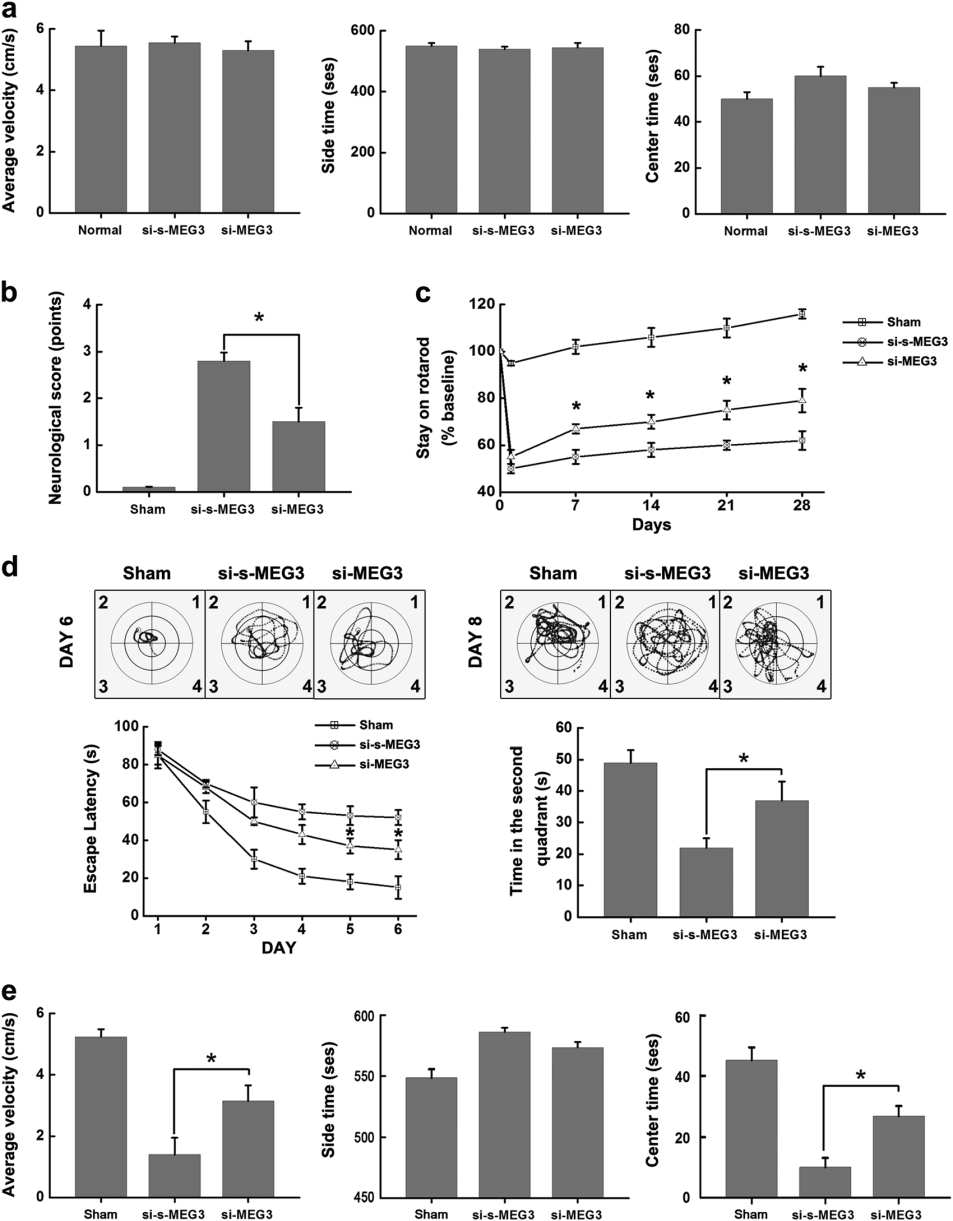
Knockdown of MEG3 improves overall neurological functions **a** The motion function and locomotor activity of the normal mice and mice treated with si-MEG3 or si-s-MEG3 was assessed by open-field test before MCAO operation. Quantifications of average velocity (left), time spent in the side areas (middle) and in the center square (right) were made for 10 min during each session. Data are means ± S.E.M. for 12 mice per group. *P >* 0.05 by Student’s *t*-test for all three parameters. **b** Neurologic deficit score. Neurologic deficits of si-s-MEG3 and si-MEG3 injection mice subjected to I/R 24 h or sham-treated mice were analyzed by a neurologic deficit score. Data are means ± S.E.M. for 8 mice per group. ^*^ *P* < 0.05 by Student’s *t*-test. **c** Rotarod test. The performance on rotarod of three groups (sham-treated mice, si-s-MEG3 and si-MEG3 injection mice subjected to MCAO operation) was analyzed before operation and throughout the 4-week period after operation. Data represent means ± S.E.M. for 20 mice per group. ^*^ *P* < 0.05 by Student’s *t*-test. **d** Morris water maze. The performance on Morris water maze of three groups (sham-treated mice, si-s-MEG3 and si-MEG3 injection mice subjected to MCAO operation) was analyzed 3 days after MCAO operation. Hidden platform training was carried out within 90 s at 6 consecutive days (6 sessions) with 4 trials each session. Curve lines (left) show the escape latency to find the hidden platform. After 6 consecutive days of invisible training, the mice rest for 1 day, and at day 8, a probe test was conducted. Bar graph shows the time spent in the target quadrant (the second quadrant). Data represent means ± S.E.M. for 20 mice per group. ^*^ *P* < 0.05 by Student’s *t*-test. **e** Open-field test. The performance on open-field test of three groups (sham-treated mice, si-s-MEG3 and si-MEG3 injection mice subjected to MCAO operation) was analyzed 3 days after MCAO operation. Data represent means ± S.E.M. for 12 mice per group. ^*^ *P* < 0.05 by Student’s *t*-test

Finally, to extend our analysis of neurological functions, we examined whether selectively knocking down of MEG3 in brains produced the therapeutic effects in mice responding to I/R. A series of behavioral analysis and a neurological scoring system were performed to evaluate the motion function and neurological deficits before and after I/R (Fig. 6a). To exclude the effect of si-s-MEG3 or si-MEG3 injection on normal neurological function, we first compared the motion function and locomotor activity in the normal mice and mice treated with si-MEG3 or si-s-MEG3 using open-field test. There was no significant difference among the three groups (Fig. 7a). Based on this, neurological status was assessed according to a neurologic deficit score following ischemia at 24 h reperfusion (Fig. 7b). We found a better neurological score in si-MEG3 injection mice compared with si-s-MEG3 (Fig. 7b). It was also noteworthy that si-MEG3 injection resulted in better recovery of motor coordination in rotarod test throughout the 4-week observation period after MCAO (Fig. 7c). Furthermore, the classic Morris water maze test was used to determine the spatial learning and memory 3 days after MCAO operation. Long-term learning and memory deficits were significantly improved in si-MEG3 treatment mice after MCAO, as manifested by an decreased latency to find the hidden platform (spatial learning ability) and increased time spent in the target quadrant (the second quadrant) when the platform was removed (spatial memory ability) compared to si-s-MEG3 treatment mice (Fig. 7d). In open-field test, we also assessed the motion function and anxiety level of si-MEG3 treatment mice by measuring their average velocity and stay time in the center area of open-field apparatus 3 days after MCAO operation. In this assessment, mice treated with si-MEG3 showed a higher velocity and spent more time in the center area of the apparatus than mice treated with si-s-MEG3, indicating an improved locomotor activity and anxiety-like behavior (Fig. 7e). Taken together, knockdown of MEG3 improves overall neurological functions *in vivo*.

## Discussion

In recent years, ncRNAs have become the major focus of studies in the treatment of ischemic stroke. LncRNAs act as epigenetic modifiers to regulate transcription and translation, while miRNAs directly target mRNAs to regulate post-transcriptional gene expression^4^. Moreover, lncRNAs could share common miRNA binding sites of mRNAs, thus blocking the binding of target mRNAs to miRNAs and abolishing the downstream effects of these miRNAs^25^. In our previous study, we first determined the expression and function of lncRNA MEG3 in cerebral ischemia^24^. However, although the relationship of MEG3 expression with a variety of tumors has been confirmed^20–23^, MEG3 have not been functionally characterized in ischemia, thus, the mechanisms underlying the effects of MEG3 in ischemia still needs to be explored deeply.

Immediately following ischemia, the energy dependent processes necessary for cells are hindered with the depletion of oxygen and glucose, and hence causes a series of biological processes^26,27^. Subsequently, a large amount of cells die as a result of necrosis, apoptosis, or both^28^. With bioinformatics, we identified that the cell death promoter MEG3 target miR-21 and hence might play an important role in ischemic neuronal death. miR-21 is a widespread expression of miRNA in mammalian cells located on chromosome 17q23.2 in humans, whose upregulation is associated with various types of solid tumors^29–31^. As for ischemia, miR-21 has been shown to be a strong anti-apoptosis and pro-survival factor, which could downregulate the expression of apoptosis related proteins^32,33^. In this study, we observed decreased expression of miR-21 following I/R or OGD/R, and overexpression of miR-21 protects OGD/R-induced apoptotic cell death, which was opposite to MEG3. These results suggested the expressional and functional negative correlation between MEG3 and miR-21 in response to ischemia.

More significantly, studies have revealed that MEG3 serves as a ceRNA for miR-21 to regulate cell proliferation or apoptosisin in some tumors^34,35^, but whether MEG3 competes with mRNA for miR-21 in ischemia and contributes to ischemic neuronal death remained unexplored. In present study, we found MEG3 share common miRNA binding sites of PDCD4 mRNA. PDCD4 is one of the known targets of miR-21. Existing evidence revealed that miR-21/PDCD4 pathway exerts anti-apoptotic effects against cell death in various diseases^36^. For example, miR-21 protects cardiomyocytes against H_2_O_2_ induced cell apoptosis by targeting the proapoptotic proteins PDCD4^37^. Additionally, miRNA-21 protects spinal cords against ischemia-reperfusion injury by inhibition of PDCD4^38^. Our results also supported this fact, moreover, we further confirmed that miR-21/PDCD4 pathway were regulated by MEG3. In the present study, MEG3 affects PDCD4 expression through downregulation of the miR-21 level and contradicts the inhibitory effects of miR-21 on PDCD4 to abolish the protection effect of miR-21 in ischemic neuronal death. So, it is the first report to determine the interaction of MEG3–miR-21–PDCD4 in ischemic stroke.

Accordingly, above data seems to point to the potential of MEG3–miR-21–PDCD4 interaction in the treatment of cerebral ischemia. To extend our analysis of neuronal protection, MEG3 was knocked down by injection of si-MEG3 with the* in vivo* transfection reagent into the lateral ventricle of mice before I/R. An important question is whether the si-MEG3 or si-s-MEG3 injection cause unexpected side effects on neurological function. Fortunately, it has been found that motion function and locomotor activity were not altered in mice treated with si-MEG3 or si-s-MEG3. Following the ischemic injury and subsequent reperfusion, we found si-MEG3 has the capability to protect against I/R-induced damage *in vivo* and shown that si-MEG3 significantly improved I/R-induced brain infract volume and profoundly reduced neuronal death. On the other hand, si-MEG3 injection revealed a better neurological score and recovery of motor coordination in mice after I/R. And what’s more, si-MEG3 treatment markedly improved long-term learning and memory deficits, locomotor activity and anxiety-like behavior post-ischemia. Thus, knockdown of MEG3 protects against ischemic damage and neurological deficits.

In conclusion, we represents the first report on a novel mechanism of lncRNA MEG3 as a ceRNA by targeting miR-21/PDCD4 signaling pathway to regulate ischemic neuronal death. Knockdown of MEG3 enhanced the protection effects of miR-21/PDCD4 pathway and prevents the followed ischemic brain injury. Therefore, targeting MEG3–miR-21–PDCD4 interaction will aid the design of new strategies for the therapeutic interventions in cerebral ischemic stroke. Nevertheless, several limitations to this study should also be acknowledged. Although here we described that MEG3 competed with PDCD4 mRNA for binding to miR-21 in ischemia, we didn’t exclude the possibility of other targets of MEG3 or miR-21 which may also contribute to the ischemic neuronal death. Since PDCD4 is not the only target of miR-21, similarly, miR-21 is not the only target of MEG3, some other targets of MEG3/miR-21 should be further analysed in the further experiments. Besides, we previously confirmed that MEG3 activated p53-mediated transactivation to mediate neuronal death in cerebral ischemia. Whether the targets of MEG3/miR-21 have a crosstalk will be analyzed in future studies.

## Materials and Methods

### Animals

Adult (90 ± 2 days of age, 25–28 g weight) male C57BL/6 J mice were used for MCAO operation. The use and care of animals were performed in accordance with the guidelines for the Care and Use of Laboratory Animals of Wuhan University and approved by the Institutional Animal Care and Use Committee. Mice were housed individually in a regulated environment of humidity and temperature (12-h light/dark cycle, lights on at 08: 00) with standard mouse diet and water.

### Luciferase reporter assay

Before luciferase reporter assay, we constructed luciferase reporters including pmirGLO-MEG3-WT, diverse mutant MEG3 (pmirGLO-MEG3-MUT1, pmirGLO-MEG3-MUT2, pmirGLO-MEG3-MUT3, pmirGLO-MEG3-MUT), pmirGLO-PDCD4-WT and pmirGLO-PDCD4-MUT. miR-21 mimics, mimic control, miR-21 inhibitor and a negative control of scramble RNA are from GenePharma (GenePharma, Shanghai, China). MEG3-expressing plasmids were constructed using the full-length sequence of MEG3 and cloned into a pcDNA3.0 vector as previously reported^24^. Si-MEG3 and si-s-MEG3 were designed as previously reported^24^. Luciferase activity was measured when cells were harvested 48 h post-transfection with the Dual Luciferase Reporter Assay System (Promega, WI, USA) according to the manufacturer’s instructions.

### RNA pulldown assay based on MS2-MBP

To further verify the relationship between MEG3 and miR-21, we performed RNA pulldown assay based on MS2-MBP (also known as MBP-affinity purification). The MS2-MBP protein was expressed and purified from E. coli. Three MS2 coat protein-binding sites (hairpins) were introduced into the downstream of MEG3 by site-directed mutagenesis to generate MEG3-WT-MS2 or MEG3-MUT-MS2 expression plasmid. N2a cells were transfected with MEG3-WT-MS2 or MEG3-MUT-MS2 and harvested 48 h post-transfection. Cells transfected with the empty vector (MS2) were used as a control. Then, cells were subjected to RNA pulldown analysis as described elsewhere. The miRNAs associated with MEG3 were confirmed by qRT-PCR analysis.

### RNA extraction and qRT-PCR analysis

Total RNA and miRNAs were extracted from brain infarction tissues in the ischemic core (Fig. 2b), or brain tissues after si-MEG3/si-s-MEG3 injection (Fig. 6b), or cultured N2a cells (Figs. 1c, 2c, 3a, 4b, and 5) by using TRIzol reagent (Invitrogen, Carlsbad, CA, USA) and miRNeasy Mini Kit (Qiagen, Hilden, Germany), respectively. For miRNA analysis, cDNA was obtained using the TaqMan MicroRNA Reverse Transcription Kit (Applied Biosystems, Foster City, CA, USA) and qRT-PCR was performed using TaqMan miRNA assay kit (Applied Biosystems). U6 small nuclear RNA (U6 snRNA) was used as an endogenous control for normalization. For mRNA analysis, cDNA was synthesized by using M-MLV reverse transcriptase (Invitrogen) and reverse transcription primers Oligo(dT). qRT-PCR was then performed with SYBR Green Real-Time PCR Master Mixes (ThermoFisher, Waltham, MA, USA) on a 7900HT Fast RealTime PCR machine (Applied Biosystems). The primers sequence were used as follows:

MEG3 forward, 5′-CTGCCCATCTACACCTCACG-3′;

MEG3 reverse, 5′-CTCTCCGCCGTCTGCGCTAGGGGCT-3′;

PDCD4 forward, 5′-CCTGAATTAGCACTGGATACTCCT-3′;

PDCD4 reverse, 5′-CTAGCCTGCACACAATCTACAGTT-3′;

GAPDH forward, 5′-GTCAACGGATTTGGTCTGTATT-3′;

GAPDH reverse, 5′-AGTCTTCTGGGTGGCAGTGAT-3′;

miR-21 stem-loop RT primer,

5′-CTCAACTGGTGTCGTGGAGTCGGCAATTCAGTTGAGTCAACATC-3′;

miR-21 forward, 5′-ACACTCCAGCTGGCTAGCTTATCAGACTGATG-3′;

miR-21 reverse, 5′-CTCAACTGGTGTCGTGGA-3′;

U6 stem-loop RT primer, 5′-AACGCTTCACGAATTTGCGT-3′;

U6 forward, 5′-CTCGCTTCGGCAGCACA-3′;

U6 reverse, 5′-AACGCTTCACGAATTTGCGT-3′.

### Focal cerebral ischemia and reperfusion model

Focal cerebral ischemia and reperfusion was induced by intraluminal MCAO as described previously^24^. Before operation, mice were anesthetized with pentobarbital sodium (30 mg/kg). Then the animal was placed on a heating panel which maintained at 37 °C during operation. Under the operating microscope, a midline incision was made for exposing the right common carotid artery, external carotid artery (ECA), and internal carotid artery (ICA). MCAO was induced by advancing a 6/0 surgical nylon monofilament with a rounded tip into the lumen of ICA gently from the right ECA until the rounded tip blocked the origin of the middle cerebral artery (MCA). After 60 min of MCAO, the surgical nylon monofilament was gently retracted to allow reperfusion for various durations (24, 48, and 72 h). The sham-operated animals were treated with similar operations except without the surgical nylon monofilament insertion.

### TTC staining and infarct volume measurement

Mice were euthanized and brains were removed quickly to froze at −25 °C for 20 min after different reperfusion time (24, 48, and 72 h) or sham operations. Then, the brain tissues were sliced into serial coronal sections (2 mm apart) and stained with 2% TTC (Sigma-Aldrich, St. Louis, MO, USA) for 30 min at 37 °C. The infract area were unstained by TTC compared with normal tissues and measured with ImageJ software (National Institutes of Health, Bethesda, MD, USA). The infarct volume in each slice was calculated by multiplying the sum of the infarct area by the slice thickness (2 mm). The percentage of infarct volume was calculated as follows: (volume of infarcted tissues of lesioned side/volume of contralateral side) × 100%.

### N2a cell culture and OGD/R model

N2a cells were cultured in Dulbecco’s Modified Eagle’s Medium (DMEM) medium (Invitrogen) supplemented with 10% fetal bovine serum (FBS, Invitrogen), 2 mM glutamine (Invitrogen), 100 μg/ml streptomycin (Invitrogen) and 100 U/ml penicillin (Invitrogen) at 37 °C with 5% CO_2_. When the cell density was appropriate, N2a cells were challenged with OGD by replacing the medium with deoxygenated glucose-free Hanks’ Balanced Salt Solution (Invitrogen). Then the cultures were transferred to a hypoxic chamber containing 5% CO_2_ and 95% N_2_ at 37 °C for 3 h. After that, the cultures were subjected to reoxygenation by exchanging the medium to glucose-containing medium plus 10% FBS and maintained under normoxic conditions for 24, 48, and 72 h at 37 °C with 5% CO_2_, respectively.

### TUNEL assay

TUNEL assay was performed using DeadEnd™ Fluorometric TUNEL system (Promega, Madison, WI, USA) according to the manufacturer’s instructions. In brief, N2a cells were fixed with 4% formaldehyde in PBS and then incubated in TUNEL reaction mixture in dark for 1 h at 37 °C. Labeled samples were visualized with a fluorescence microscope (OLYMPUS IX71, Shinjuku-ku, Tokyo, Japan) and counted using Image J software (National Institutes of Health).

### Western blot assay

Total proteins were extracted from cells or tissues with RIPA lysis buffer (Sigma-Aldrich) on ice. Protein concentration were separated by sodium dodecyl sulfate polyacrylamide gel (SDS-PAGE) electrophoresis (BioRad, Hercules, CA, USA) and transferred onto nitrocellulose membranes (Millipore). Blocking was performed in 5% non-fat milk for 1 h at 37 °C. After that, membranes were incubated with anti-rabbit primary antibody against PDCD4 (Sigma-Aldrich) at a 1: 1000 dilution overnight at 4 °C. β-actin served as an endogenous control and detected using a rabbit polyclonal at 1:4000 dilution (Abcam, Cambridge, MA, USA). The following day, The secondary antibody of goat anti-rabbit IgG at a 1: 1000 dilution (Santa Cruz Biotechnology, Santa Cruz, CA, USA) was incubated for 1 h at room temperature. After washing, signals were visualized using LI-COR Odyssey Imaging System (LI-COR, Lincoln, NE, USA).

### Stereotaxic injection of siRNA in mice

Mice were anesthetized and fixed to a stereotaxic apparatus (Stoelting, Kiel, WI, USA). A total of 5 μl si-MEG3 or si-s-MEG3 was diluted with the same volume of *in vivo* transfection reagent (Entranster^TM^-*in vivo*; Engreen, Beijing, China) and administered into the lateral ventricle of mice. The stereotaxic coordinates were as follows: 0.6 mm posterior to the bregma, 1.0 mm lateral to the midline, and 2.5 mm under the dura. One day post injection, MCAO operation was established.

### Fluoro-Jade C staining

3 days following ischemia, mice were anesthetized and perfused. The brain frozen sections (30 μm) were dried overnight for FJ staining. First, the slices were immersed for 5 min in 80% ethanol supplemented with 1% sodium hydroxide, for 2 min in 70% ethanol, for 10 min in 0.06% potassium permanganate and washed for 1 min in distilled water. Second, sections were incubated for 20 min in 0.01% FJ (Millipore) solution dissolved in 0.1% acetic acid. Images were acquired by epifluorescence microscopy (OLYMPUS IX71). The number of FJ-positive cells (FJ^+^) was measured using ImageJ software (National Institutes of Health).

### Open-field test

Motion function and locomotor activity were evaluated in a clear chamber measuring 43.2 cm × 43.2 cm, which was outfitted with a video-tracking system (EthoVision XT 8.0; Noldus Technology, Wageningen, The Netherlands) for monitoring horizontal and vertical activity of mice. Mice were placed in a corner of the open-field apparatus and allowed to explore for 10 min. Then, the average velocity and time spent in the center square or side was measured.

### Neurological status assessment

Neurological status was assessed according to a neurologic deficit score following ischemia at 24 h reperfusion. Neurological deficit score: 0 = no observable neurological deficits; 1 = failure to extend left forepaw; 2 = circling to the left; 3 = falling to the left; 4 = cannot walk spontaneously.

### Rotarod test

To assess motor coordination and learning before and after I/R, an accelerating rotarod test was performed (SD Instruments, San Diego, CA, USA). Mice were placed on the rungs of the rotarod with rotation speed increasing from 2 to 25 rpm over 2 min. A trial ended if the mice fell off the rungs, or gripped and spun around for one revolution without attempting to walk on the rungs. The animals were acclimatized to the rotarod for 5 trials daily for 3 days before injection of si-MEG3 or si-s-MEG3. Follow-up tests were performed at 1, 7, 14, 21, 28 days after MCAO. The data are expressed as the mean duration (in seconds) per day compared with the measurements 1 day before injection.

### Morris water maze

The water maze task consisted a circle water tank (120 cm-diameter and 60 cm high) filled with opaque water (21–23 °C) and a round platform (6 cm-diameter) which was submerged 1 cm beneath the surface of water at the center of the second quadrant. Before the start of hidden platform training, mice were allowed to acclimate to the testing environment for 30 min. Hidden platform training was carried out within 90 s at 6 consecutive days (six sessions) with four trials each session. If the mice failed to find the invisible platform within 90 s, the researcher would lead the mice to the platform and allowed the mice to stand on it for 15 s. Escape latency to find the hidden platform, swimming paths, swimming velocity were recorded. After 6 consecutive days of invisible training, the mice will rest for 1 day, and at day 8, a probe test was conducted. The platform was removed and mice was allowed to search the pool for 90 s. The time spent in each quadrant was analyzed. Data was traced by a TM-Vision video-tracking system (Chengdu Taimeng Software Co. LTD, Chengdu, Sichuan Province, China).

### Statistical analysis

Statistical analysis was performed with GraphPad Prism 5.01 software (GraphPad Software, La Jolla, CA, USA). *P*-value < 0.05 was considered to have statistically significant difference (^*^ *P* < 0.05; ^**^ *P* < 0.01).
